# Lost in Translation: Ambiguity in Nerve Sheath Tumor Nomenclature and Its Resultant Treatment Effect

**DOI:** 10.3390/cancers5020519

**Published:** 2013-05-08

**Authors:** Nicholas M. Bernthal, Kevin B. Jones, Michael J. Monument, Ting Liu, David Viskochil, R. Lor Randall

**Affiliations:** 1 Sarcoma Services, Department of Orthopaedics, Huntsman Cancer Institute and Primary Childrens Medical Center, University of Utah, Salt Lake City, UT 84112, USA; 2 Department of Pathology, Huntsman Cancer Institute, University of Utah, Salt Lake City, UT 84112, USA; 3 Division of Medical Genetics, Department of Pediatrics, University of Utah, Salt Lake City, UT 84112, USA

**Keywords:** MPNST, neurofibromatosis, nerve sheath tumor, atypical neurofibroma, low grade MPNST

## Abstract

There is much ambiguity surrounding the diagnosis of nerve sheath tumors, including atypical neurofibroma and low-grade MPNST, and yet, the distinction between these entities designates either benign or malignant behavior and thus carries presumed profound prognostic importance that often guides treatment. This study reviews the diagnostic criteria used to designate atypical neurofibroma from low-grade MPNSTs and reviews existing literature the natural history of each of these tumors to see if the distinction is, in fact, of importance.

## 1. Introduction

Peripheral nerve sheath tumors (PNSTs) comprise a spectrum of neoplastic potential ranging from benign neurofibromas and schwannomas to high-grade malignant peripheral nerve sheath tumors (MPNSTs). Benign neurofibromas are often found incidentally and usually require only symptomatic treatment. In contrast, high-grade MPNSTs are fulminantly malignant lesions with clinical outcomes on par with the worst of soft tissue sarcomas, despite aggressive treatment. Along this spectrum, atypical neurofibromas and low-grade MPNSTs reside in an undefined, nebulous middle ground that presents a treatment challenge to physicians and surgeons. While convention defines atypical neurofibromas as benign lesions and low-grade MPNSTs as malignant, this distinction may not be so clear. In this analysis, we review the present literature on atypical neurofibromas and low-grade MPNSTs to evaluate the clinical utility of this distinction. In doing so, we hope to help guide treatment for these challenging tumors.

## 2. Methods

A PUBMED search was performed on 20 September 2012, searching for the following key words: “atypical neurofibroma”, “malignant peripheral nerve sheath tumor”, “low-grade MPNST”, and “neurofibromatosis”. Articles from 1970–2012 were reviewed. 

## 3. A Rose by Any Other Name [1]

### 3.1. Neurofibroma

**Figure 1:**
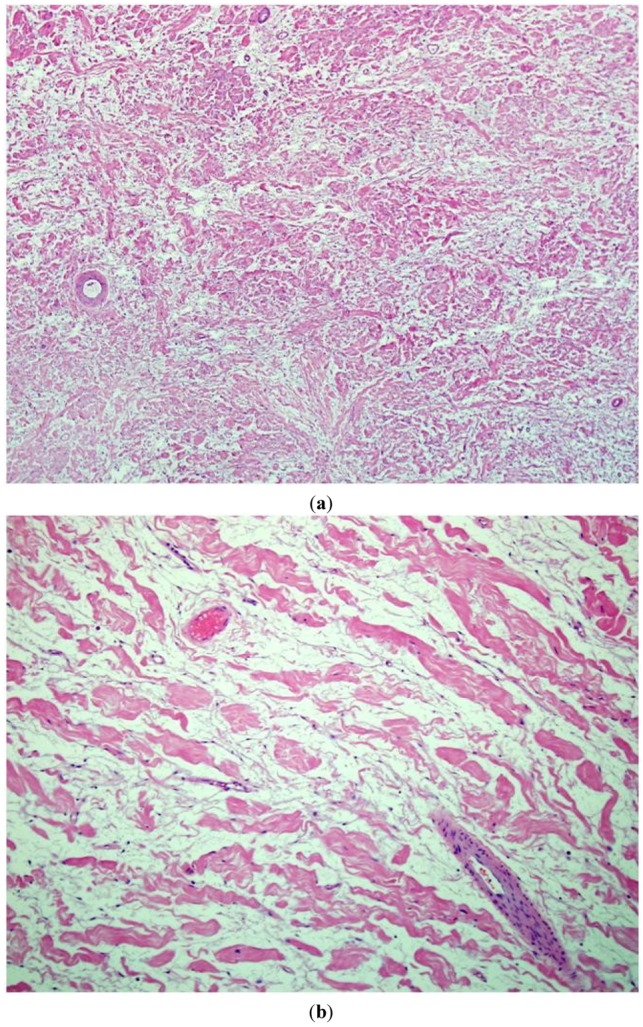
An intermediate (200×) (**a**) and high power view (400×); (**b**) of routine hematoxylin and eosin (H&E stain) sections of typical neurofibromas from an NF1 patient. A low cellularity of spindly (Schwann) cells intermixed with abundant stromamucin and prominent “shredded carrot” collagen. There is no evidence of cytologicatypia, increased cellularity, or mitotic figures.

According to the World Health Organization (WHO), a neurofibroma is an unencapsulated nerve sheath tumor comprised of a heterogenous population of cells of mixed origin in which Schwann cells are the predominant cell type [2]. Neurofibromas are the most common type of peripheral nerve sheath tumor and approximately 10% are associated with the genetic disorder neurofibromatosis type 1 (NF1) [3]. Neurofibromas are often described as “benign, slow-growing, and often painless” and treatment is predominantly surgical resection of those tumors that are symptomatic [4]. Neurofibromas are often subclassified into localized, diffuse and plexiform types based on their gross appearance and location [2]. Morphologically, neurofibromas are generally described as having mild nuclear size and hyperchromasia, minimal mitotic activity and no areas of necrosis [5]. The spindly cells show wavy, small and dark nuclei without cytological atypia. Intermixed are dense, wire-like collagen, often described as “shredded carrot” type. A myxoid or mucin rich stroma matrix is noted (Figure 1a,b). 

### 3.2. Malignant Peripheral Nerve Sheath Tumors (MPNSTs)

**Figure 2:**
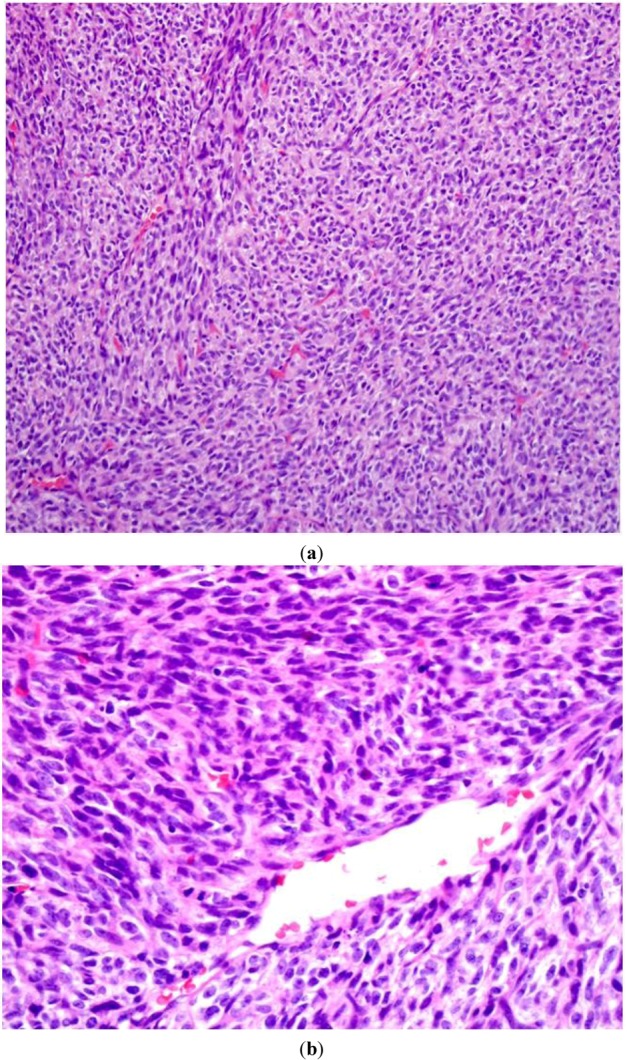
An intermediate power (200×) (**a**) view of routine hematoxylin and eosin (H&E stain) sections of a high-grade MPNST from an NF1 patient. A high cellularity of spindly (Schwann) cells intermixed with a fascicular growth pattern and little “shredded carrot” collagen. High power (400×); (**b**) view shows increased cellularity, atypia, and mitotic figures.

MPNSTs are defined by the WHO as “any malignant tumor arising from a peripheral nerve or showing nerve sheath differentiation, excluding those originating from epineurium or peripheral nerve vasculature” [2]. This broad definition encompasses tumors previously classified as neurogenic sarcoma, malignant schwannoma, malignant neurilemoma, and neurofibrosarcoma [4]. While several variants have been described including epithelioid, glandular, and rhabdomyosarcomatous (triton tumor), high-grade MPNSTs are generally thought to grow rapidly, cause severe pain, and lead to “relentlessly progressive neurologic deficits” [4]. In contrast to neurofibromas, MPNSTs are histologically characterized by a fascicular arrangement of grossly atypical wavy spindle cells with an increased nuclear:cytoplasm ratio, a high mitotic index, minimal “shredded carrot” type collagen, high mitotic activity, a fascicular growth pattern, and prominent areas of necrosis [5] (Figure 2a,b). 

## 4. Shades of Grey

Unfortunately for physicians and patients alike, the aforementioned pathohistological criteria used to differentiate neurofibroma from MPNST are most effective at differentiating “typical” neurofibromas from high-grade MPNSTs. However, the intermediate lesions, the atypical neurofibromas and low-grade MPNSTs, have characteristics of both tumors, and therefore present a difficult diagnostic and treatment conundrum [6]. 

### 4.1. Atypical Neurofibroma

An atypical neurofibroma is classified as such based on increased cellularity, more degenerative cytological atypia, and a more pronounced fascicular growth pattern as compared to the more “typical” neurofibroma. While all of these characteristics are more suggestive of MPNST, atypical neurofibromas lack the “monotonous” cytological atypia, chromatin abnormalities and mitotic activity seen in MPNST [5]. 

### 4.2. Low-Grade MPNST

An MPNST is histologically defined as a hypercellular tumor with nuclear enlargement, hyperchromasia, and a pronounced fascicular growth pattern (similar to atypical neurofibroma). A low-grade MPNST is one in which less than five mitoses per 10 high-power fields are noted in the setting of the aforementioned signs of malignancy [5]. Further, necrosis is conspicuously lacking.

## 5. Imaging

Positron-emission/computerized tomography (PET/CT) has been applied to help distinguish benign and malignant nerve sheath tumors [7]. The mean standardized uptake value (SUV) in benign neurofibroma has been calculated in one series at 1.5 (SD 1.1) as compared to 5.7 (SD 2.6) in MPNST [8]. However, again the intermediate lesions muddy the waters: recent work applying the aforementioned scale to determine malignancy shows an atypical neurofibroma in a patient with NF1 with a high, “malignant-level” SUV of 5.7 on PET/CT [9]. 

## 6. Choose Wisely…

The rather ambiguous classification noted above, distinguishing atypical neurofibroma from low-grade MPNST is an ostensibly critical one in guiding treatment. A low-grade MPNST is, by definition, a malignant lesion, and therefore convention suggests treatment with wide resection in an attempt for oncologic cure. Conversely, an atypical neurofibroma, a benign lesion, can be treated with intralesional or marginal resection if a wide margin carries significant morbidity. With this major threshold-initiated shift in treatment paradigms in mind, getting the classification “correct” becomes of utmost significance. 

The situation becomes more complex when one examines the clinical behavior of nerve sheath tumors. While treatment is often guided reflexively by the categorizing of a tumor as benign or malignant, the natural history of low-grade MPNSTs and of atypical neurofibromas must be considered. In other words, do low grade MPNSTs truly exhibit malignant behavior and are atypical neurofibromas actually benign? This question drives the present review. 

## 7. Clinical Outcomes

Studies looking at MPNSTs often aggregate low- and high-grade lesions into one category of “Malignant Peripheral Nerve Sheath Tumor.” This grouping clearly skews clinical results to assign a more “malignant” course for low-grade MPNSTs than may be justified. Similarly, studies looking at neurofibromas often include both typical and atypical neurofibromas, thereby raising the possibility of “diluting” a more aggressive natural history of atypical neurofibromas. We therefore reviewed existing literature on low-grade MPNSTs and atypical neurofibromas to see if these lesions are clinically distinguishable at two separate intermediately aggressive groups.

The largest study to date of MPNSTs is a single-institution review of 205 patients with localized MPNST treated with surgery at the Nazionale per lo Studio e la Cura dei Tumori in Milan, Italy [10]. While the report’s discussion concludes that tumor grade is not a significant factor for survival, close analysis of the data brings this conclusion into doubt. For the statistical analysis, the authors combine low and intermediate grade MPNSTs as a single group, then compare these to high-grade tumors, concluding that the groups do not differ in survival. Intermediate grade MPNST in the French Grading system used in this cohort includes tumors with a total score of 4–5, which could include a tumor that is completely undifferentiated (3 points) and has 10–19 mitoses per 10 HPF (2 points) [11]. Such a so-called intermediate grade tumor has more in common histologically with a high-grade MPNST than a low-grade MPNST. Such blurred distinctions may well distort a survival comparison. When low-grade tumors are reviewed as an independent group, only 6% of the cohort, or 14 total patients, can be considered. One of these 14 patients (7%) developed local recurrence and metastatic disease, ultimately dying of disease. The authors did not designate whether this patient had NF1, which might indicate development of a second MPNST, rather than a true recurrence of the first tumor.

A recent publication from the Mayo Clinic reviewed the institution’s experience with 175 MPNST patients [12]. This study classifies MPNSTs on a four-tier grading system, with grade 1 and 2 tumors (well differentiated and intermediately differentiated) as “low grade” and grade 3 and 4 tumors as “high grade.” They conclude that tumor grade was significantly correlated with disease specific survival on uni- and multi-variate analysis. The 50 patients with grade 1 or 2 “low grade” MPNSTs included seven who developed metastatic disease (14%). This again includes “intermediately differentiated” tumors as low-grade but similar to the Italian cohort, cannot rule out the possibility of aggressive behavior in “low-grade” lesions. 

Unfortunately, the remaining literature addressing outcomes of low-grade MPNSTs is limited to small case series. Okada *et al.* published a series of 55 cases of MPNST, in which six were classified as low-grade by AJCC classification [13]. All six host patients showed no evidence of disease at final follow-up after resection. As a diagnostic aside, the authors note that all low-grade MPNSTs were S100 positive in their cohort whereas only 50% of high-grade MPNSTs demonstrated S100 positivity. Yamaguchi *et al.* published a retrospective case series of four cases of low-grade MPNSTs (AJCC classification) [14]. In this study, there were no recurrences, metastases or deaths attributable to disease in these patients with low-grade tumors. 

These four studies represent the complete literature available for low-grade MPNSTs, paltry as it is. Atypical neurofibromas have even less conclusive data available than low-grade MPNSTs. The term, “atypical neurofibroma” is most often associated with a neurofibroma in the setting of a patient with NF1 and has recently been described by several studies as a “premalignant lesion in transit”, *i.e.*, in the early stages of transitioning from a benign neurofibroma to an aggressive MPNST. While some cytogenetic analyses were recently published providing support for this hypothesis [15], there are no published articles currently available in PUBMED showing clinical/oncologic outcomes of atypical neurofibromas.

## 8. Discussion

The spectrum of neoplastic potential ranging from benign neurofibromas to MPNSTs continues to challenge our diagnostic and therapeutic capabilities today. Between the biological extremes—a benign neurofibroma producing only local symptoms and a high-grade MPNST progressing rapidly to metastasis and death despite aggressive treatment—most lesions lie somewhere in the middle range. Evidence for a correlation between histologic features and ultimate clinical course is rather weak at any level, but downright absent for the graded distinctions between atypical neurofibromas and low-grade MPNSTs.

This ambiguity challenges the patient and the treating physician. Tailored treatment options along a range of aggressiveness are not really available. We basically have non-operative surveillance, marginal, nerve-sparing excisions, and wide, morbid resections in our arsenal. Because ultimately high-grade tumors are so profoundly recalcitrant to any treatment options, any tumor *known* to be on a biological course headed that direction would merit aggressive and morbid treatment while it is yet possible. Conversely, any tumor *known* to be stable in a non-progressive state would be happily observed over time, or symptomatically managed with a minimally-morbid resection. The distinction is of paramount importance, but remains woefully unclear. The question is highlighted in NF1 patients, in whom “atypical” neurofibromas often track along the nerve sheath of an entire extremity all the way to the nerve roots at the spinal cord. Massively disfiguring and debilitating surgeries are often performed on these patients in the name of oncologic principles with no clear information available regarding their biology or natural history. 

Future work aims to address this ambiguity in two ways. First, the science of molecular genetics is rapidly advancing and may provide us a better understanding of what truly defines a “malignant” nerve sheath tumor. Recent cytogenetic data suggests that mutations in the *CDKN2A* and *CDKN2B* loci, which code for cell cycle regulators p16 and p14, accumulate as part of the “process” by which neurofibromas become malignant [15]. Other work suggests that MPNSTs arise from precursors via silencing mutations in multiple tumor suppressor genes (e.g., *TP53*, *CDKN2A*) and amplifications of tyrosine kinase receptor genes (e.g., *EGFR*) [16,17,18,19]. A recent case report presents genomic changes in one area of a neurofibroma that “de-differentiated” to a MPNST, where losses of TP53, RB1, and CDKN2A were found only in the malignant portion of the tumor [20]. Additional work has been done attempting to correlate MDM2 amplification with p53 immunoreactivity to better distinguish among nerve sheath tumors, but with little proposed current applicability [21]. Nonetheless, future molecular diagnostic analyses will undoubtedly assist us to define the lesions more accurately than we are currently able to with histopathology and immunohistochemistry. 

The second area for future work lies in well-organized clinical outcome studies. Our institution, among others, is in the process of analyzing the clinical outcomes of atypical neurofibromas and low-grade MPNSTs. With widely agreed upon criteria and a common grading system, large-scale, multi-institutional studies could more clearly define the discernible risk factors (histopathologic or molecular) for a bad prognosis meriting more aggressive treatment. 

## 9. Conclusions

While the distinction among nerve sheath tumors remains diagnostically challenging with current criteria, the importance of the distinction in determining the malignant potential of these tumors remains unclear in the current literature. Previous clinical studies have used inconsistent definitions and diagnostic criteria; thus, coalescing current data into a meta-analysis is not feasible. Molecular genetics will hopefully provide more reproducible definitions of these intermediate lesions and therefore allow more accurate assessment of natural history. This is essential prior to determining the appropriateness of aggressive and often debilitating surgical procedures.
